# Inhibition of the ATP synthase sensitizes *Staphylococcus aureus* towards human antimicrobial peptides

**DOI:** 10.1038/s41598-020-68146-4

**Published:** 2020-07-09

**Authors:** Liping Liu, Christian Beck, Katrine Nøhr-Meldgaard, Andreas Peschel, Dorothee Kretschmer, Hanne Ingmer, Martin Vestergaard

**Affiliations:** 1 Department of Veterinary and Animal Sciences, Faculty of Health and Medical Sciences, University of Copenhagen, Stigbøjlen 4, 1870 Frederiksberg C, Denmark; 2 Department of Infection Biology, Interfaculty Institute for Microbiology and Infection Medicine Tübingen (IMIT), Cluster of Excellence ‘Controlling Microbes to Fight Infections’, University of Tübingen, Auf der Morgenstelle 28, 72076 Tübingen, Germany

**Keywords:** Microbiology, Antimicrobials, Antimicrobial resistance

## Abstract

Antimicrobial peptides (AMPs) are an important part of the human innate immune system for protection against bacterial infections, however the AMPs display varying degrees of activity against *Staphylococcus aureus*. Previously, we showed that inactivation of the ATP synthase sensitizes *S. aureus* towards the AMP antibiotic class of polymyxins. Here we wondered if the ATP synthase similarly is needed for tolerance towards various human AMPs, including human β-defensins (hBD1-4), LL-37 and histatin 5. Importantly, we find that the ATP synthase mutant (*atpA*) is more susceptible to killing by hBD4, hBD2, LL-37 and histatin 5 than wild type cells, while no changes in susceptibility was detected for hBD3 and hBD1. Administration of the ATP synthase inhibitor, resveratrol, sensitizes *S. aureus* towards hBD4-mediated killing. Neutrophils rely on AMPs and reactive oxygen molecules to eliminate bacteria and the *atpA* mutant is more susceptible to killing by neutrophils than the WT, even when the oxidative burst is inhibited.These results show that the staphylococcal ATP synthase enhance tolerance of *S. aureus* towards some human AMPs and this indicates that inhibition of the ATP synthase may be explored as a new therapeutic strategy that sensitizes *S. aureus* to naturally occurring AMPs of the innate immune system.

## Introduction

Bacterial pathogens that cause disease in humans remain a serious threat to public health and antibiotics are still our primary weapons in treating many bacterial diseases. The ability to eradicate bacterial infections is however challenged by development of resistance for every type of antibiotic introduced to the clinic^1^. The majority of the new small molecule antibiotics in clinical development are however inhibiting the same targets as already marketed antibiotics^2^. As an alternative to small molecule antibiotics, antimicrobial peptides (AMPs) are also explored in clinical trials, however most of the AMPs are only tested for topical applications due to toxicity issues and low metabolic stability^3^. Here we propose a new strategy to combat bacterial infections, namely to sensitize bacteria to the naturally occurring antimicrobial peptides of the human body and hence boosting the antibacterial capabilities of the innate immune system to eradicate bacterial infections.

Humans are continuously exposed to numerous, and potentially pathogenic, microorganisms, where the innate immune system provides the first line of defense. AMPs constitute an important defense mechanism of the innate immune system against invading microorganisms, due to their antimicrobial and immune stimulatory properties^4,5^. In humans, several classes of AMPs have been identified, such as α- and β-defensins, the cathelicidin LL-37 and histatins^5^. The α-defensins consist of six members, which are divided into human neutrophil peptides (HNP1-4) and human α-defensin 5 and 6 (HD5 and HD6). HNP1-4 are highly concentrated in the granules of neutrophils, but are also expressed in monocytes, lymphocytes and natural killer cells. HD5 and HD6 are primarily expressed in Paneth cells of the small intestine^4^. The β-defensins consist of four members (hBD1-4) and are primarily secreted by mucosal surface epithelia, e.g. by keratinocytes in the human skin^6^. Histatins comprise a family of cationic, histidine-rich peptides that are present in human saliva and are important for maintaining oral health by limiting infections in the oral cavity^5^. Several histatins have been characterized, however histatin 5 displays the strongest antimicrobial activity^5^. LL-37 is an antimicrobial peptide that belongs to the cathelicidin family and is expressed in various epithelial- (e.g. keratinocytes) and immune cells (e.g. neutrophils and macrophages)^7^. The bactericidal activities of many AMPs have generally been attributed to pore formation in bacterial cytoplasmic membranes, however this mode of action may be too simplistic^8,9^. The bactericidal activity of hBD3 has for example been associated with lipid II binding, leading to inhibition of cell wall biosynthesis^10^ and some AMPs also have intracellular targets^11^. Many antimicrobial peptides display a net positive charge, which is important in the initial electrostatic attraction to negatively charged bacterial phospholipid membranes and negatively charged teichoic acids on the surface of Gram positive bacteria, e.g. *Staphylococcus aureus*^11^.

*S. aureus* is a common colonizer of the human body^6^, where approximately 30–50% of healthy adults transiently carry this species and approximately 20% are persistently colonized^12^. The skin, nose and intestinal tract are important ecological niches for *S. aureus* carriage^13^. Topical colonization with *S. aureus* imposes a risk for subsequent infections, if the skin or mucosal barriers are breached and enables transmission of *S. aureus* cells to the adjacent tissues or the bloodstream^12^. *S. aureus* is an opportunistic pathogen that may cause life-threatening diseases, such as sepsis, endocarditis and pneumonia^12^. Even though keratinocytes express various antimicrobial peptides, such as hBD1-4 and LL-37, *S. aureus* frequently colonizes human skin^6^. Among the human β-defensins, only hBD3 displays potent bactericidal activity against *S. aureus* at physiological conditions^14–16^, and keratinocytes are dependent on hBD3 for killing of *S. aureus*^17^. However, it is incompletely understood, why the remaining β-defensins display limited anti-staphylococcal activity. This indicates that sensitizing *S. aureus* towards the innate immune system AMPs may potentially facilitate eradication of colonizing *S. aureus*.

Multiple factors affect bacterial susceptibility towards AMPs, such as cell membrane composition, cell surface charge and transmembrane potential^8^. The positive charge of many AMPs facilitates the interaction with negatively charged bacterial envelopes^11^. A common resistance mechanism exploited by bacteria is to reduce the net negative charge of the cell envelope, for example by lysinylation of phospholipids^18^ and D-alanylation of teichoic acids in *S. aureus*^19^. Curiously, deficiency of wall teichoic acids selectively confers reduced susceptibility to hBD3, while not affecting susceptibility to LL-37 and HNP1-3^20^. The transmembrane potential affects the ability of cationic AMPs to permeabilize membranes^8,21^, where an inside-negative transmembrane potential facilitates insertion of some cationic AMPs into bacterial membranes^8^. Interference with the electron transport chain by inactivation of menaquinone- (*men* mutants) or hemin (*hem* mutants) biosynthesis pathways leads to membrane depolarization in *S. aureus* and in the appearance as small colony variants (SCVs) on agar plates^22^. Electron transport chain SCVs have been associated with reduced susceptibility towards multiple AMPs, including hBD2-3^23^, thrombin-induced platelet microbicidal protein^24^ and nisin^24^. We recently reported that inactivation of genes encoding for multiple subunits of the ATP synthase sensitizes *S. aureus* towards polymyxins^25^, a class of cationic AMPs that is used for treatment of Gram-negative infections^26^. The ATP synthase basically serves two physiological functions, first being synthesis of ATP from ADP and inorganic phosphate by using energy from the proton motive force. Secondly, during conditions with a low proton-motive force the ATP synthase can work in reverse as an ATPase and thereby contributes to the establishment of a cross-membrane proton gradient through ATP hydrolysis^27^. In *S. aureus*, the ATP synthase is primarily used to hydrolyze ATP for maintaining the cross-membrane proton gradient both under fermentative and respiratory conditions^28^. ATP synthase inactivation in *S. aureus* leads to hyper-polarization of the membrane^25,28^, which was hypothesized to be the mechanism for sensitizing ATP synthase mutants towards polymyxins^25^.

Several molecules have been shown to inhibit the ATP synthase in different species^29^. For example inhibition of the ATP synthase with oligomycin A sensitizes *S. aureus* towards polymyxin B^25^ and aminoglycosides^30^. However, oligomycin A displays similar 50% inhibitory concentration (IC_50_) between *S. aureus* and human mitochondrial ATP synthases^31^, and cannot be used clinically due to toxicity issues^32^. Resveratrol is a widely used nutraceutical that has been shown to bind to the bovine ATP synthase in the F_1_-domain^33^ and also binds reversibly to the ATP synthase in *E. coli*, partially inhibiting both ATP hydrolysis and ATP synthesis^34^. Co-administration of resveratrol sensitizes *S. aureus* towards aminoglycosides^30^. However, resveratrol is readily metabolized following oral administration, which probably only enables topical use^35^.

Since ATP synthase inactivation sensitizes *S. aureus* towards polymyxins, we hypothesize that this strategy also can sensitize *S. aureus* towards various human AMPs. Potentiation of human AMPs that are currently ineffective against *S. aureus* may potentially become a new therapeutic strategy, where treatment relies on deprivation of AMP resistance mechanisms and hence boosting of the naturally occurring AMPs of the innate immune system.

## Materials and Methods

### Bacterial strains, growth conditions and chemicals

The *Staphylococcus aureus* JE2 wild type (WT) strain and derivative mutants used in this study are highlighted in Table 1. Antimicrobial peptides used in this study included histatin-5 (Innovagen, Sweden), LL-37 (Isca Biochemicals, United Kingdom) and hBD1-4 (Innovagen, Sweden), as well as polymyxin B Etests (bioMérieux, France). We used the ATP synthase inhibitor resveratrol (Santa Cruz Biotechnology). Bacterial strains were routinely cultured at 37 °C in tryptic soy broth (TSB) or on tryptic soy agar (TSA).

**Table 1:** Strains and mutants used.

Organism	Description and genotype	Source
*S. aureus*	JE2, CA-MRSA USA300	
*S. aureus*	JE2 *menD*::ΦNΣ	^60^
*S. aureus*	JE2 *atpA*::ΦNΣ	^60^
*S. aureus*	JE2 *atpB*::ΦNΣ	^60^
*S. aureus*	JE2 *atpG*::ΦNΣ	^60^
*S. aureus*	*atpA*^+^—Strain with allelic exchange of the transposon insertion (*atpA*::ΦNΣ) with the intact *atpA* gene	^25^

### Antimicrobial susceptibility assays

#### Microdilution

The minimum inhibitory concentration for resveratrol was determined using a two-fold broth microdilution assay in TSB (100 µl) with an initial inoculum of approximately 5 × 10^5^ cells/ml. MIC was determined upon incubation at 37 °C for 24 h.

#### Etest

The MIC for polymyxin B was determined using Etest (bioMérieux, France) in the absence and in the presence of sub-inhibitory concentrations (0.0625x–0.25 × MIC) of resveratrol or menadione (1 µg/ml, Sigma). From overnight cultures, strains were diluted to approximately 10^8^ CFU/ml and then distributed on TSA plates using a sterile cotton swab. MIC was determined upon incubation at 37 °C for 24 h.

#### Bacterial cell survival assays

From overnight cultures of *S. aureus* JE2 and mutants, 10 μl was diluted into 990 μl fresh TSB medium in a falcon tube and grown for 2 h for the cells to reach early exponential phase. After 2 h the cultures were re-suspended in 10 mM sodium phosphate buffer (pH 7.4), termed NaPi (Medicago, Sweden). Cells were subsequently diluted in NaPi to approximately 5 × 10^5^ CFU/ml, and combined with antimicrobial peptides and resveratrol when indicated, to a final volume of 100 µl and incubated in 96-well plates with shaking for 2 h at 37 °C. Bacteria were plated for CFU on TSA plates. Following overnight incubation at 37 °C for 24 h, viable cells were enumerated and relative cell survival was calculated as CFU_with peptide_/CFU_without peptide_ at 2 h post-exposure. Values provided are the mean ± SEM derived from at least three independent biological replicates.

### Isolation of PMNs from human blood

Blood was collected from healthy adult volunteers and written informed consent was given. Isolation of neutrophils was performed following the procedure described in^36^. All methods were carried out in accordance with relevant guidelines and regulations. The institutional review board (IRB) of the University of Tübingen approved the study and all adult subjects provided informed consent. This study was done in accordance with the ethics committee of the medical faculty of the University of Tübingen that approved the study, Approval number 015/2014 BO2. Briefly, heparinized blood was diluted 1:1 (v/v) with PBS containing 0.5% BSA and 2 mM EDTA and layered onto a gradient of Biocoll (density, 1.077 g/ml; Biochrom) and Histopaque (density, 1.119 g/ml; Sigma). After centrifugation for 20 min at 380×*g*, neutrophils were collected from the Histopaque phase. Cells were subjected to a brief hypotonic shock with ultrapure-water containing 155 mM ammonium chloride, 1 mM potassium hydrogen carbonate and 0.1 mM EDTA at pH 7.4, washed, and suspended at 2.5 × 10^6^ cells/ml in RPMI containing 200 mg/ml HSA, 2 mM glutamine, 2 mM sodium pyruvate and 10 mM HEPES.

### Phagocytosis of *S. aureus* by neutrophils

Starter cultures of *S. aureus* JE2 and its respective *atpA* mutant were grown in TSB medium overnight. Main cultures were subsequently inoculated at an OD_600_ of 0.1 and grown to an OD of 1. 10^9^ CFU of WT and *atpA* mutant cells were adjusted in PBS, stained with 10 µM carboxyfluorescein succinimidyl ester (CFSE) for 1 h at 37 °C and subsequently washed 3 times with PBS. 10^8^ CFU/ml were opsonized with 10% normal human serum (NHS) in RPMI for 1 h at 37 °C. To check for correct CFUs, the dilution was plated onto TSA agar plates. 2.5 × 10^6^ previously isolated PMNs were seeded in a 96 well round-bottom plate and challenged with opsonized WT and *atpA* mutant *S. aureus* JE2 at a MOI of 1. Incubation was performed for 20 min or 1 h at 37 °C. After incubation, the plate was centrifuged at 300×*g* for 10 min, and the pellet was fixed with 3.7% formaldehyde for 20 min in the dark. The fixed cells were then washed and suspended in PBS. The samples were analyzed with a BD FACSCalibur by measuring 5000 events for each sample.

### Killing of *S. aureus* by neutrophils

Starter cultures of *S. aureus* JE2 and its respective *atpA* mutant were grown in TSB medium overnight. Main cultures were subsequently inoculated at an OD_600_ of 0.1 and grown to an OD of 1. 10^9^ CFU of WT and *atpA* mutant cells were adjusted in PBS medium, then washed and resuspended in PBS. 10^8^ CFU/ml were opsonized with 10% normal human serum (NHS) in RPMI for 1 h at 37 °C. 2.5 × 10^6^ previously isolated PMNs were seeded in 96 well round-bottom plates and challenged with opsonized WT and *atpA* mutant *S. aureus* JE2 at a MOI of 1. Incubation was performed for 1 h at 37 °C in a 96 well round-bottom plate and inoculation controls were included. After incubation, the plate was centrifuged at 300×*g* for 10 min, the supernatants were collected, and the remaining neutrophil pellets were lysed using cold ddH_2_0 for 10 min on a rocker. The lysed neutrophils and remaining bacteria were resuspended and pooled with the previously collected supernatants. Dilutions of 10^–2^ were plated on TSA plates with an Eddy Jet 2 W and incubated overnight at 37 °C. Values provided are the mean ± SEM derived from at least seven independent biological replicates.

### Statistics

The data were analyzed in GraphPad Prism 7 (GraphPad Software Inc.) using paired t-tests or one-way analysis of variance (ANOVA) with a post hoc analysis of Dunnett’s multiple comparison tests. Log-transformed data was used for bacterial survival. *P* < 0.05 (*), *P* < 0.01 (**) and *P* < 0.001 (***).

## Results

### ATP synthase mutants are more susceptible to hBD2 and hBD4

**Figure 1:**
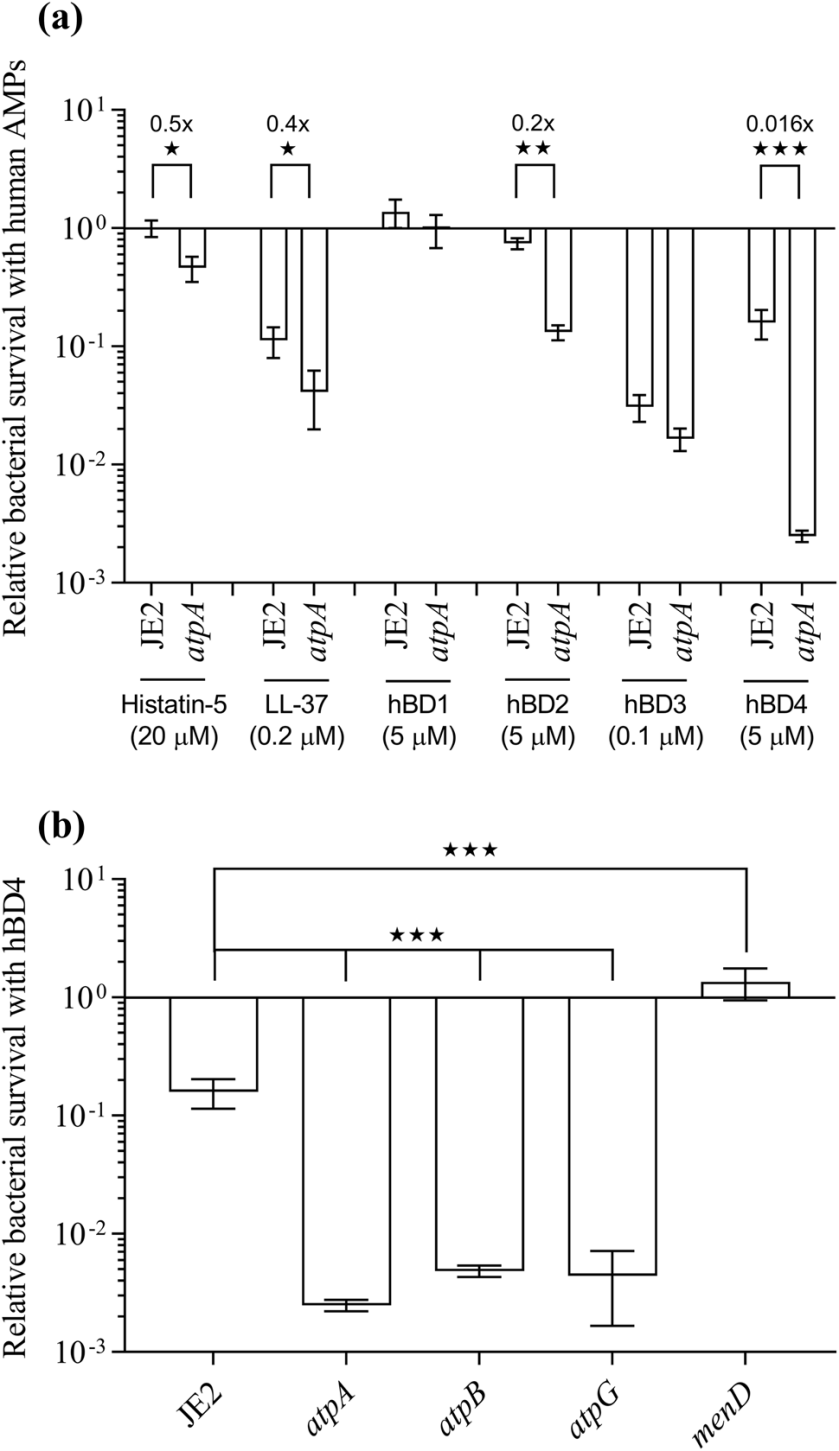
ATP synthase mutants are more susceptible to specific human AMPs than the WT. (**a**) The susceptibilities to the different AMPs assayed are presented as the relative survival following 2 h exposure at the indicated AMP concentrations for JE2 (WT) and *atpA* mutant. (**b**) Survival of ATP synthase mutants (*atpA*, *atpB* and *atpG*) and *menD* mutant following 2 h exposure to hBD4 (5 µM). Each survival value provided is the mean ± SEM derived from at least three independent measurements. ★*p* < 0.05, ★★*p* < 0.01 and ★★★*p* < 0.001.

Since inactivation of the ATP synthase increases susceptibility of *S. aureus* towards the antimicrobial peptides, polymyxins^25^, we wondered if inactivation of the ATP synthase also sensitizes *S. aureus* towards AMPs of the human innate immune system. Therefore, bacterial killing of the WT *S. aureus* JE2 and isogenic *atpA* (ATP synthase subunit alpha) transposon mutant was assessed following 2 h exposure to human cationic AMPs, comprising histatin-5, LL-37 and hBD1-4 at the concentrations highlighted in Fig. 1a. The *atpA* mutant was more susceptible to hBD4 and hBD2 compared with the WT (Fig. 1a). The *atpA* mutant displayed a 63-fold greater reduction in viable cells compared with WT upon treatment with hBD4. For hBD2, the *atpA* mutant displayed a fivefold greater reduction in viable cells compared with WT. A minor increase in bactericidal activity against the *atpA* mutant was detected for histatin-5 and LL-37, whereas no differences between WT and *atpA* survival were detected upon treatment with hBD1 nor hBD3.

We assessed hBD4-mediated killing of other ATP synthase mutants, namely *atpB* (ATP synthase subunit A) and *atpG* (ATP synthase subunit gamma) and both mutants displayed increased susceptibility to hBD4, similarly to the *atpA* mutant (Fig. 1b).

Since the *atpA* mutant has a hyperpolarized membrane^25^, we also assessed hBD4 susceptibility of the *menD* transposon mutant, which has a depolarized membrane^37^. The *menD* mutant was indeed more tolerant to hBD4 compared with WT, as no reduction in viable cell count was observed following 2 h exposure to hBD4 at 5 µM (Fig. 1b), suggesting that the magnitude of the membrane potential is an important determinant for hBD4 susceptibility.

Similarly we observed this correlation between magnitude of membrane potential and polymyxin B susceptibility, where the *menD* mutant was more resistant towards polymyxin B compared with the WT (Supplementary Table S1). The *menD* mutant is auxotrophic for menadione and supplementation with the compound re-sensitized the *menD* mutant to polymyxin B (Supplementary Table S1).

Taken together, inactivation of the ATP synthase sensitizes *S. aureus* to specific human AMPs and the magnitude of the membrane potential correlates with hBD4 susceptibility. This correlation also applies more broadly to include the non-human AMP, polymyxin B.

### The ATP synthase inhibitor resveratrol sensitizes *S. aureus* towards hBD4

**Figure 2:**
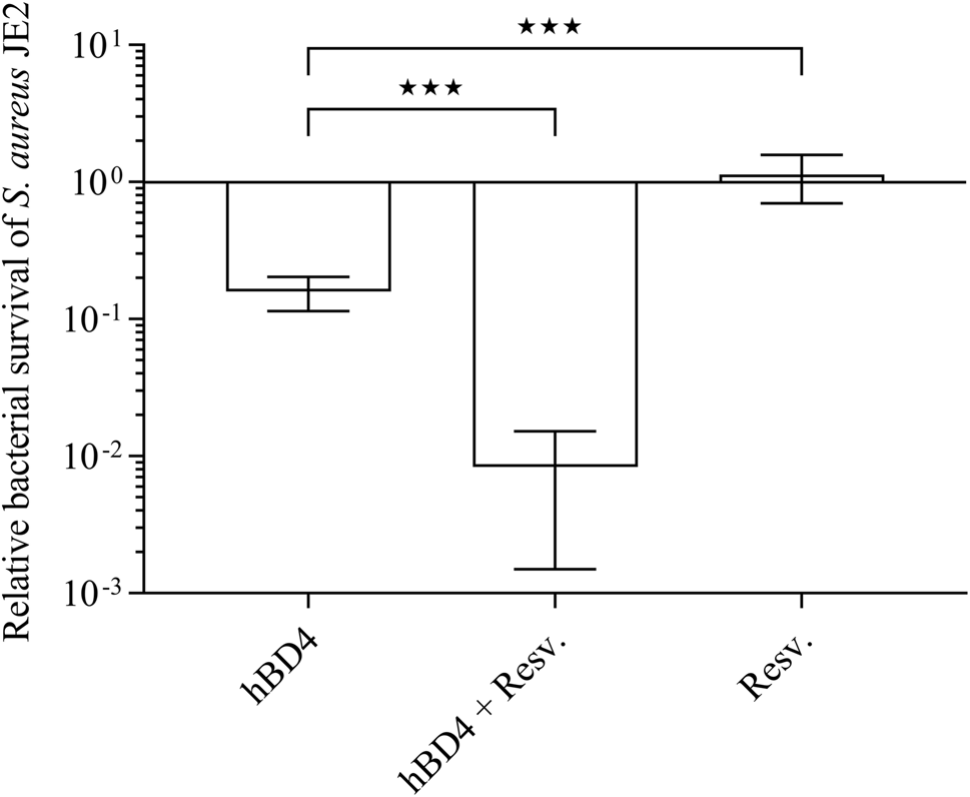
Resveratrol sensitizes *S. aureus* to hBD4. Survival of *S. aureus* JE2 was assessed for resveratrol (32 µg/ml) and hBD4 (5 µM), either alone or in combination. Each value provided is the mean ± SEM derived from at least three independent measurements. ★*p* < 0.05, ★★*p* < 0.01 and ★★★*p* < 0.001.

Resveratrol is a putative ATP synthase inhibitor in *S. aureus*^30^ and therefore, we assessed if resveratrol could sensitize *S. aureus* JE2 towards hBD4. Resveratrol has growth-inhibitory properties with a MIC of 256 µg/ml, but at a sub-inhibitory concentration (0.125 × MIC) it had no impact on *S. aureus* viability (Fig. 2). Importantly, however, when supplemented in combination with hBD4, resveratrol increased killing of WT *S. aureus* by 20-fold compared with hBD4 alone.

Similarly, supplementation of resveratrol at sub-inhibitory concentrations (0.0625x–0.25 × MIC) sensitized *S. aureus* JE2 to polymyxin B (Supplementary Table S2).

These result suggests that ATP synthase inhibition with resveratrol may be an attractive approach to sensitize *S. aureus* towards hBD4.

### The *atpA* mutant is more susceptible to killing by human neutrophils

**Figure 3:**
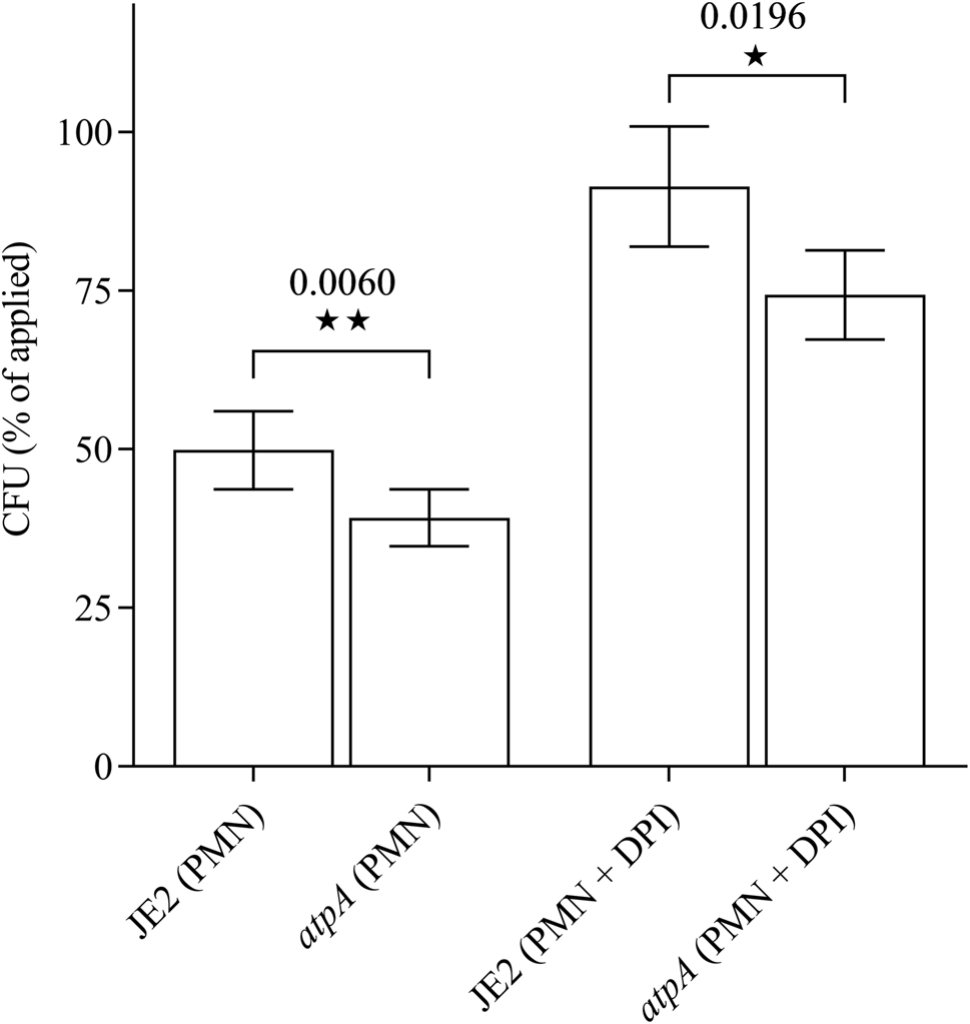
Neutrophil-mediated killing of *S. aureus*. The percentage of viable opsonized WT and *atpA* mutant cells following incubation with neutrophils (PMN) for 1 h. Surviving cells are expressed in percentage of the initial counts. Diphenyleneiodonium (DPI) is a NADPH oxidase inhibitor. Each value provided is the mean ± SEM derived from at least seven independent measurements. ★*p* < 0.05, ★★*p* < 0.01 and ★★★*p* < 0.001.

Log-phase bacteria of the WT and *atpA* mutant were opsonized with pooled normal human serum, phagocytosed by neutrophils, and subsequently incubated for one hour before determination of surviving cells. The *atpA* mutant was more susceptible to neutrophil killing than the WT and after one hour of incubation with neutrophils, only 39.2% of the *atpA* cells survived compared with 49.9% for the WT (*P* = 0.006) (Fig. 3). The uptake of the strains into the neutrophils was similar (Data not shown), suggesting that the increased killing of the *atpA* is due to antimicrobial activities of the neutrophils and not due to alterations in phagocytosis rates.

As neutrophils normally use both, oxygen-dependent and non-oxygen-dependent killing mechanisms, including antimicrobial peptides^38^, we compared survival of *atpA* and WT in neutrophils treated with the NADPH oxidase inhibitor diphenyleneiodonium (DPI), which suppresses the formation of reactive oxygen species^18^. Suppression of the oxidative burst limited killing of *S. aureus* (Fig. 3). After one hour incubation only 74.3% of the *atpA* cells survived in DPI-treated neutrophils, compared to 91.4% for the WT (*P* = 0.0196) (Fig. 3). These data suggest that the *atpA* mutant is more susceptible towards the oxygen-independent antimicrobial activities of neutrophils.

## Discussion

Antimicrobial peptides are an important part of the innate immune system and the AMPs display activity against a wide range of bacterial-, fungal- and viral species^6^. Several human AMPs however display low inhibitory activity against *S. aureus*^14^. For example, the human β-defensins 1–4 are produced by keratinocytes and are key in protecting against skin infections^6^. hBD3 displays greater bactericidal activity against *S. aureus* than the other β-defensins^14–16^, and hBD3 is important for keratinocytes in killing *S. aureus*^17^. Production of AMPs in the skin and in the nasal passages plays a major role in preventing *S. aureus* persistent colonization and people with defects in hBD3 production have enhanced nasal colonization of *S. aureus*^39^. Our results point to a novel type of antimicrobial therapy, whereby the susceptibility of the pathogen is enhanced towards the natural human antimicrobial peptides. Here we demonstrate the potential for *S. aureus*, but it may be applicable to other human pathogens as well.

The energetic state of bacterial membranes can affect the susceptibility towards AMPs in different bacterial species^8^ and for some conventional classes of antibiotics as well, i.e. aminoglycosides^40^. Recently, we demonstrated that ATP synthase mutants of *S. aureus* become more sensitive towards polymyxins^25^. ATP synthase inactivation confers hyperpolarization of the membrane^25,28^ and larger membrane potentials can facilitate AMP insertion into membranes^8^. In this study, we demonstrate that the activities of certain human AMPs are affected by the magnitude of the membrane potential. ATP synthase mutants have a hyperpolarized membrane^25,28^ and become more sensitive towards hBD2 and hBD4 and to a minor degree towards LL-37 and histatin-5 (Fig. 1a). Contrarily, a *menD* mutant with a depolarized membrane is less sensitive towards hBD4 (Fig. 1b).

Our finding that membrane depolarization protects *S. aureus* from hBD4-mediated killing corroborate previous studies demonstrating that *S. aureus* SCVs are less susceptible to different AMPs. For *S. aureus*, electron-transport chain deficient mutants are less susceptible to killing by thrombin-induced PMP-1 (tPMP-1)^41^, nisin^24^, lactoferrin B^42^ and human AMPs, including hBD2, hBD3 and LL-37^23^. Another study, with genetically defined *menD* and *hemB* mutants in different *S. aureus* genetic backgrounds did however not observe changes in susceptibility to LL-37^43^. Killing by the human neutrophil defensin 1 (hNP-1) is similar in WT and mutants with impaired electron transport chains^24,41,44^. These observations indicate that membrane potential changes only affect the activity of specific AMPs.

It has been suggested that membrane depolarization and subsequently increased tolerance towards AMPs of the innate immune system is a survival strategy that enable intracellular persistence of *S. aureus* in eukaryotic cells^45^. Here we demonstrate that inactivation of the ATP synthase contrarily sensitizes *S. aureus* to neutrophil-killing (Fig. 3). The increased susceptibility to neutrophil-killing is also evident when the oxidative burst is suppressed (Fig. 3), suggesting that this effect is mediated by increased susceptibility to AMPs produced by neutrophils^38^.

It is not only in *S. aureus* that AMP sensitivity is modulated by the magnitude of the membrane potential. In *E. coli*, inactivation of the ATP synthase also leads to hyperpolarization of the membrane^46^ and ATP synthase mutants are more sensitive to colistin^47^ and aminoglycosides^40,47^. Deletion of the gene *phoP* in *E. coli* conferred hyperpolarization of the membrane and a concomitant increase in activity of polymyxin B, while collapsing the proton gradient with the protonophore carbonyl cyanide *m*-chlorophenyl hydrazone (CCCP) abrogated this effect^48^. Dissipation of membrane potential with CCCP also impaired killing of *E. coli* with the AMP indolicidin^49^. For *Salmonella enterica* Typhimurium, impairment of the electron transport chain reduces AMP activity, e.g. a *hemB* mutant displays a fourfold increase in MIC for colistin^50^. Even respiration-deficient mutants of the fungus *Candida albicans* experience reduced sensitivity to histatin-5 and chemical inhibition of the electron transport chain with sodium azide or CCCP treatment also protects *C. albicans* against histatin-5 killing^51,52^.

The ATP synthase may potentially be targeted to facilitate killing by AMPs of the innate immune system and hence be essential under in vivo conditions. By employing the Tn-seq methodology, the ATP synthase has been identified in several studies as essential during in vivo conditions, such as in abscess formation or osteomyelitis, while the ATP synthase is dispensable during growth in laboratory medium^53–55^. Recently, Grosser and colleagues demonstrated that an ATP synthase mutant indeed is severely attenuated in a murine skin abscess model^28^. ATP synthase inactivation confers pleiotropic effects, including attenuated growth under anaerobic conditions, increased sensitivity towards peroxide and nitric oxide stresses^28^. Whether virulence attenuation of the ATP synthase mutant in the murine skin abscess model is mediated by a single phenotypic trait or a combination thereof remain unexplored in the study^28^ and here we provide an additional phenotype that may contribute to the attenuated virulence, namely increased sensitivity of *S. aureus* to different AMPs of the innate immune system.

Inhibition of the ATP synthase may potentially have therapeutic value either as a monotherapy or in combination with AMPs or aminoglycosides. Many ATP synthase inhibitors have been identified^29^, however several of these, e.g. oligomycin A, are not selective for bacterial ATP synthases and also blocks human mitochondrial ATP synthases^31^. The ATP synthase has been clinically validated as a druggable target in recent years with the antibiotic bedaquiline that selectively inhibits ATP synthases of Mycobacteria^56^. We demonstrated that the ATP synthase inhibitor resveratrol, a commonly used nutraceutical^57^, sensitizes *S. aureus* towards hBD4 (Fig. 2). Resveratrol has previously been assessed for clearance of acne skin lesions^58^ and has recently been shown to reduce abscess formation by *S. aureus* when used as a monotherapy^59^. It will be important in future animal experiments to elucidate, whether resveratrol in combination with human defensins are superior in treating topical *S. aureus* infections compared with the respective monotherapies.

It is encouraging that bacterial ATP synthases are sufficiently different from human ATP synthases, which enables identification of selective bacterial ATP synthase inhibitors that are not toxic to human cells^32^. Selective staphylococcal ATP synthase inhibitors may provide a novel class of antibacterial therapies that is based on sensitizing *S. aureus* towards the AMPs of the innate immune system. Additionally, such inhibitors can be adjuvants that potentiate the activity of conventional antibiotics, such as aminoglycosides and polymyxins^25,30^. Several AMPs are in clinical development^3^, whose activity potentially also can be enhanced by co-administration of ATP synthase inhibitors.

In summary, we have investigated the possibility of sensitizing *S. aureus* towards human AMPs by targeting the ATP synthase and our results suggest that it may be a novel strategy for development of new antimicrobial therapeutics.

## Supplementary information

Supplementary file1 (DOCX 15 kb)
