# Supplementary material for: Inhibition of the ATP synthase sensitizes Staphylococcus aureus towards human antimicrobial peptides
Source: Sci Rep. 2020 Jul 9;10:11391. doi: 10.1038/s41598-020-68146-4 (PMC7347559; doi:10.1038/s41598-020-68146-4)
Supplement: Supplementary file 1 — Supplementary file1 (DOCX 15 kb) [file 41598_2020_68146_MOESM1_ESM.docx]

Supplementary Information

# **Inhibition of the ATP synthase sensitizes *Staphylococcus aureus* towards human antimicrobial peptides**

Liping Liu^1^, Christian Beck^2^, Katrine Nøhr-Meldgaard^1^, Andreas Peschel^2^, Dorothee Kretschmer^2^, Hanne Ingmer^1*^ & Martin Vestergaard^1^

1. Department of Veterinary and Animal Sciences, Faculty of Health and Medical Sciences, University of Copenhagen, Stigbøjlen 4, DK-1870 Frederiksberg C, Denmark.
2. Department of Infection Biology, Interfaculty Institute for Microbiology and Infection Medicine Tübingen (IMIT), University of Tübingen, Auf der Morgenstelle 28, 72076 Tübingen, Germany.

**Supplementary Table S1** – MIC of polymyxin B against *S. aureus* and derivative mutants.

| Strain and condition | MIC (µg/ml) |
| --- | --- |
| *S. aureus* JE2 | 128 |
| *atpA*::ΦNΣ | 16 |
| *atpA^+^* | 96 |
| *menD*::ΦNΣ | > 1024 |
| *menD*::ΦNΣ + menadione | 192 |

**Supplementary Table S2** – Resveratrol at sub-inhibitory concentrations sensitizes *S. aureus* to polymyxin B.

| [Resveratrol] | Polymyxin B MIC (µg/ml) |
| --- | --- |
| TSA + 0 µg/ml resveratrol | 128 |
| TSA + 16 µg/ml resveratrol | 64-96 |
| TSA + 32 µg/ml resveratrol | 48 |
| TSA + 64 µg/ml resveratrol | 24 |
